# A Trust-Aware Architecture for Personalized Digital Health: Integrating Blueprint Personas and Ontology-Based Reasoning

**DOI:** 10.1007/s10916-025-02255-3

**Published:** 2025-10-04

**Authors:** Alina Vozna, Andrea Monaldini, Stefania Costantini

**Affiliations:** 1 University of Pisa, Largo B. Pontecorvo, Pisa, 56127 Italy; 2 Department of Information Engineering, Computer Science and Mathematics, University of L’Aquila, L’Aquila, Italy

**Keywords:** Intelligent agent, Healthcare, Blueprint personas, Ontology, Trust

## Abstract

This paper presents a trust-aware architecture for personalized digital health that combines user modeling, symbolic reasoning, and adaptive trust mechanisms. The proposed system uses Blueprint Personas to capture detailed patient profiles, including clinical, behavioral, and emotional traits. These profiles guide an intelligent agent that interacts with patients and healthcare professionals to provide context-sensitive support. Personalization is achieved through an ontology-based reasoning layer that interprets user needs and integrates real-time data from electronic health records, wearable devices, and environmental sources. To promote transparency and foster long-term user engagement, the system includes a formal trust modeling component based on a Reference Ontology of Trust (ROT), allowing the system to flexibly tailor communication strategies in response to user feedback and evolving trust levels. A simulated scenario involving a patient with chronic obstructive pulmonary disease demonstrates how the system delivers proactive and personalized healthcare interventions, such as medication reminders and air quality alerts. While the architecture is modular and designed for scalability, it has not yet been deployed in real-world clinical settings. Empirical validation and integration with clinical platforms remain part of future work. Nevertheless, this ongoing work contributes to the development of explainable and ethically aligned AI systems that enhance autonomy, accessibility, and trust in digital health environments through explainable reasoning.

## Introduction

The preservation of autonomy and improving quality of life is a critical concern in various populations, not just the elderly, as people face numerous challenges due to cognitive or physical impairments. Although many people maintain the ability to make decisions, issues such as memory loss, disabilities, or chronic health conditions can complicate daily living.

Socially Assistive Robots (SARs) [1], powered by artificial intelligence (AI), offer a promising technological solution to address these challenges by helping users manage daily tasks, encouraging social interaction, and improving overall well-being [2]. By integrating AI, SARs have the potential to reduce the user’s dependency while enhancing cognitive engagement and socialization.

The goal of this project is to develop an intelligent agent capable of interacting with both users and healthcare providers in a personalized and adaptive manner [3]. Designed for deployment on cost-effective platforms, the agent aims to support individuals requiring assistance in their everyday lives. This approach is particularly relevant for managing chronic conditions and comorbidities that compromise long-term autonomy.

To maximize the benefits of robot-human interaction, it is crucial to consider how SARs can act as social catalysts. They can serve as conversation starters, encouraging meaningful exchanges between users and their peers or caregivers. For example, interaction models such as the Paro robot seal [4] have been shown to reduce stress and foster meaningful conversations among users and caregivers. These socially mediated interactions are not only functional but also emotionally supportive.

True empathy in robotic systems extends beyond functional assistance. It requires the ability to recognize and respond to users’ emotional and psychological needs [5]. For a robot to be perceived as empathetic, it must exhibit personalized behaviors that reflect an understanding of the user’s unique circumstances, preferences, and challenges. This level of personalization is key to fostering trust, engagement, and improved care outcomes. Robots can foster deeper, more meaningful interactions by integrating detailed patient information, such as medical history, social context, and personal preferences, improving the overall user experience and patient outcomes [6].

In this work, we introduce a novel architecture that integrates three core components, Blueprint Personas for user modeling, ontology-based reasoning for semantic adaptation, and the Reference Ontology of Trust (ROT) [7] to dynamically calibrate user trust. These elements are combined into a layered and extensible system architecture designed to provide ethical, transparent, and context-aware digital health support.

We focus on the case of chronic obstructive pulmonary disease (COPD) to ground our approach in a clinically relevant scenario where daily support, adaptability, and emotional sensitivity are critical. Our goal is to develop an intelligent personal assistant capable of delivering personalized and trustworthy digital health interventions, with a modular and extensible architecture applicable to a broad range of conditions.

To ensure broad accessibility and flexibility, the proposed intelligent agent is platform-agnostic and can be deployed across multiple hardware configurations. Depending on user needs and context, it may function as a voice-based application on a mobile device, as part of a smart home assistant, or as the cognitive core of a socially assistive robot. This modularity allows the system to support users with different levels of technological familiarity, including older adults and individuals with cognitive or physical impairments.

To support early-stage validation of our approach, we generated a dataset of synthetic patient profiles using a large language model (LLM), guided by structured templates derived from questionnaire variables and persona design. These simulated personas allow us to test the reasoning and personalization mechanisms of the system in a controlled, yet diverse setting, without requiring sensitive clinical data. While not based on real-world users, this methodology provides a scalable and ethical way to explore the interaction between trust, personalization, and user modeling in the healthcare domain.

The remainder of this paper is structured as follows: Section “Related Work” presents related work on user modeling, ontology-based systems in healthcare, and trust-aware AI, followed by a positioning of our contribution. Section “Blueprint Personas for Healthcare Personalization” introduces the Blueprint Personas framework and its role in personalization. Section “Questionnaire Methodology and Persona Generation” details the methodology used to generate personas from structured questionnaires. Section “System Architecture” describes the system architecture, highlighting its modular design and four-layer structure. Section “Ontologies and Trust Reasoning” explores the use of ontologies and introduces the Reference Ontology of Trust for semantic adaptation and trust calibration. Section “Application Scenario: COPD Patient Support” illustrates an application scenario involving a COPD patient, presenting the symbolic implementation of reasoning and trust adaptation. Section “Expected Evaluation and Impact” outlines the evaluation criteria and expected impact. Section “Ethical Considerations” discusses key ethical considerations. Finally, Section “Conclusion” concludes the paper and outlines future work.

## Related Work

Personalization in digital health has been shown to improve engagement, adherence, and care quality, particularly for older adults and patients with chronic conditions. Traditional systems, however, often rely on generic or static profiles, which fail to capture the complexity of patient behavior and context.

To address this, several studies have explored the use of personas as user modeling tools. LeRouge et al. emphasized the importance of conceptual user models, such as personas and user profiles, in designing consumer health technologies adapted for aging populations [8]. More recently, Ten Klooster et al. proposed the Persona Approach Twente, combining patient data from interviews, electronic health records (EHRs), and log data to construct empirically grounded eHealth personas [9].

Other contributions, such as those by Matias et al., introduced dynamic personas that evolve over time, incorporating behavior change theories (e.g., COM-B, Transtheoretical Model) to better reflect users’ psychological and social states [10]. Chute et al. further extended the use of personas by incorporating lived experience narratives and self-reported data into evolving digital health profiles. [11]. Abdullah et al. applied personas in combination with emotional goal modeling to design culturally sensitive mHealth interventions, showing how emotional and clinical attributes can be jointly encoded to enhance personalization. [12].

Despite these advances, current approaches often remain isolated from semantic reasoning and trust modeling. Our framework extends this work by integrating personas within a reasoning architecture that also incorporates ontologies and adaptive trust mechanisms.

In parallel, ontologies have gained traction as a powerful method for structuring healthcare knowledge, enabling semantic interoperability and supporting intelligent reasoning. Elhadj et al. developed Do-Care, a modular ontology-based system that integrates multiple vocabularies (e.g., FOAF, SSN/SOSA, ICNP) and employs SWRL rules to infer chronic disease states [13]. Similarly, Titi et al. proposed an ontology-based healthcare monitoring framework for IoT environments, emphasizing real-time interoperability between patient data, sensors, and contextual inputs. [14].

Ajami and Mcheick designed an ontology framework for COPD patients, incorporating environmental, physiological, and activity-related data to trigger timely interventions [15]. Other efforts, such as the eHeaRSS system by Lee and Kim, use a combination of ontology and case-based reasoning to provide patient-centered recommendations based on shared experiential knowledge [16].

However, these approaches typically prioritize structural modeling or clinical inference, often overlooking patient behavior, personalization dynamics, and adaptive trust.

Our framework addresses these limitations by unifying personas, ontological knowledge, and trust calibration in a single architecture. This integration enables real-time, personalized support while maintaining semantic transparency and ethical alignment, crucial attributes in the development of next-generation digital health systems.

### Positioning of Our Work

Compared to existing systems, our contribution lies in the explicit integration of user modeling, semantic reasoning, and dynamic trust calibration within a single symbolic framework. While previous works either focus on static profiles or employ ontologies for structuring clinical data, our system goes further by using ontological concepts directly in the agent’s reasoning process, enabling explainable and personalized adaptation in real time.

In particular, our use of ROT is not limited to documentation or conceptual modeling: we encode ROT elements as reasoning predicates within an ASP-based engine, making trust computationally actionable. This allows the agent to infer trust levels from user behavior (e.g., ignoring or following a prompt), detect trust erosion, and adapt interaction tone accordingly.

Unlike ontology-based systems such as Do-Care or eHeaRSS, which focus on structural interoperability, our approach binds ontology semantics directly to behavioral adaptation and trust management. Similarly, although some works use personas or user profiles, they do not embed them in symbolic decision-making processes, nor calibrate trust dynamically as part of the interaction loop.

This level of integration enables a layered, explainable pipeline where user data flows through semantic filters, symbolic inference, and adaptive interfaces, allowing for intelligent, ethical, and trust-sensitive digital health support. To the best of our knowledge, this is the first implementation combining Blueprint Personas, ontology-based reasoning, and runtime trust calibration in a unified, logic-based architecture.

Considering other existing proposals, in [17], review recent work on Generative Agents (GAs) powered by Large Language Models (LLMs) as a transformative addition to Agent-Based Modeling (ABM). These agents, with conversational capabilities and emergent behavior modeling, offer improved realism over traditional rule-based agents. They report that, in the ongoing work by [18], Personas are used to generate parameters that characterize a GA and hold agents’ status. Reporting from [17], these parameters typically include high-level data like age, sex, income level, race, employment, and location, together with social information detailing possible relationships between agents. This information, with the help of the extensive knowledge embedded in LLMs, can guide the decision-making process of an agent to emulate a human-like personality accurately. As a matter of fact, GAs, according to [17] appear particularly suited for simulation of social dynamics in various contexts, enabling the implementation of more realistic and adaptive human behaviors (e.g., they propose an example in the context of city simulation). In contrast, “traditional” agents, like the one that we propose, also according to [17] guarantee stability and predictability of behaviour, and produce consistent and replicable results because they rely on deterministic models that ensure agents always act according to the defined logic. Instead, GAs can introduce non-determinism and variability due to the probabilistic nature of LLMs, may hallucinate or produce unrealistic, inconsistent, or contextually irrelevant responses, and are “black-boxes”. In our context, where reliability and explainability are crucial issues, we opted for rule-based agents with well-defined behavioral mechanisms that render agents’ decision-making processes fully transparent, interpretable, and efficient. This does not exclude employing LLMs to build more natural and flexible user interfaces.

## Blueprint Personas for Healthcare Personalization

The European Blueprint on Digital Transformation of Health and Care for the Aging Society, funded as part of the WE4AHA project under the European Commission’s digital single market strategy, reflects a shared policy vision on how innovation can transform health and care provision in our aging society. The first time it was presented was in December 2016 at the EIP on AHA Conference [19]. It was described as a shared policy vision by many stakeholders, including policymakers, civil society, professional organizations, and industry, in the first update in 2017. This marks a new strategy for securing funding and commitments for the digital transformation of health and care [20].

### Why use a Blueprint Persona

Personas are archetypal user representations derived from behavioral and contextual data, enabling the design of systems that are in line with the actual goals and needs of users. Rather than relying on explicit self-reporting, personas are constructed through inference from observational and survey data, making them particularly effective in capturing latent user expectations in complex environments such as healthcare [21]. By synthesizing demographic, psychological, and behavioral traits, personas support the development of empathetic, user-centered digital services [22].

**Fig. 1:**
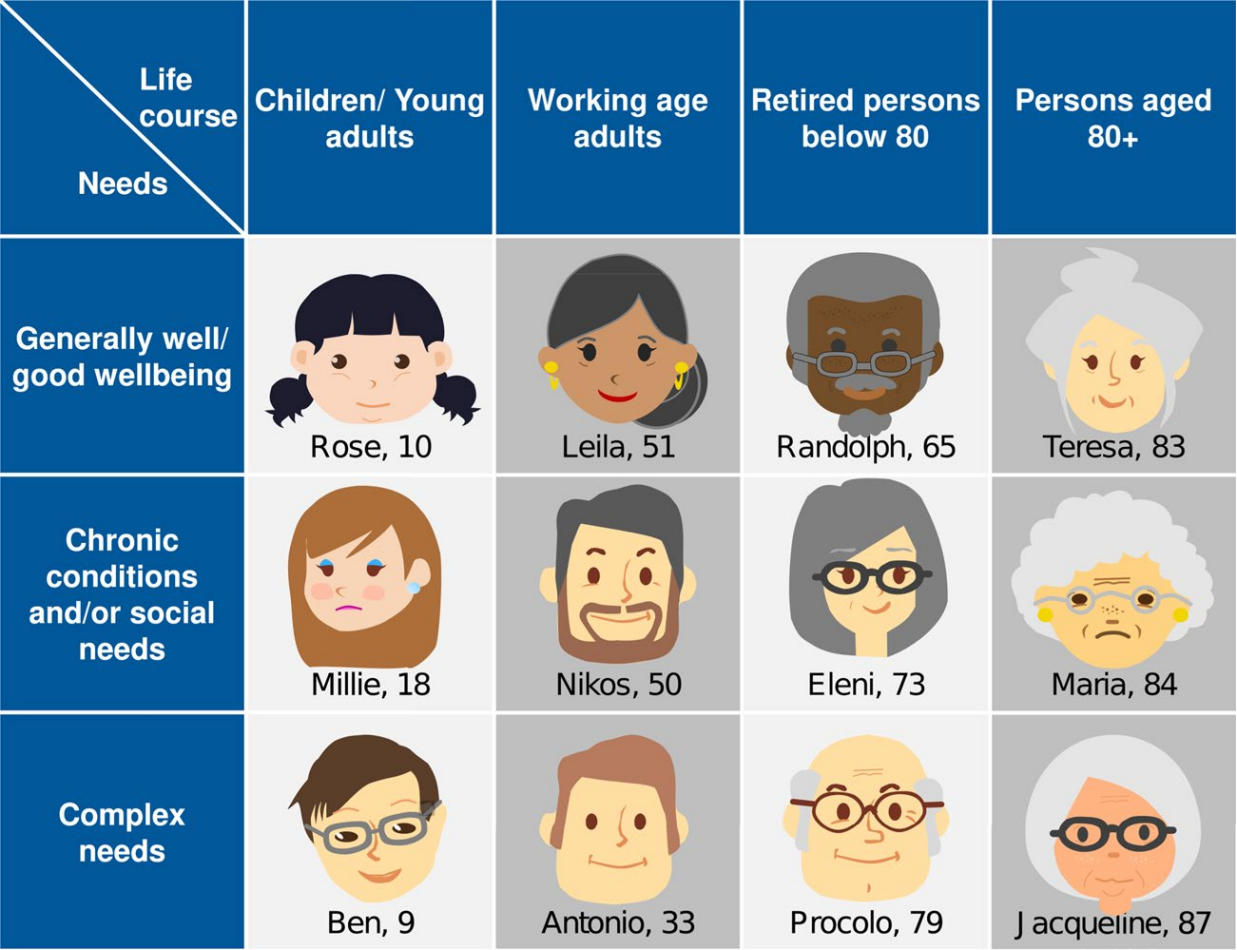
Matrix of Blueprint personas. Source: https://blueprint-personas.eu/

The Blueprint Personas framework offers a structured set of twelve reference personas representing key population segments, including children, adults, retirees, and individuals over 80, as illustrated in Fig. 1. These personas account for varying levels of health, care needs, and social complexity, offering a comprehensive basis for understanding diverse user profiles in digitally supported care contexts. They are designed to be adaptable and extensible, allowing them to be tailored to specific use cases, health conditions, and cultural settings.

In our system, Blueprint Personas are used as the foundation for adapting interactions between the intelligent agent and the patient. As a case study, we focus on patients with COPD, who present a wide range of challenges, including physiological fragility, emotional vulnerability, and limited access to digital technologies. Although the choice of COPD is not driven by disease-specific computational needs, it provides a well-defined testbed to evaluate person-centered reasoning and interaction.

The integration of personas within the agent’s architecture guides both interaction design and functional adaptation. For instance, the agent can offer personalized medication reminders, monitor relevant symptoms such as respiratory rate and coughing patterns, and support physical activity and stress reduction through contextually relevant suggestions. Emotional well-being is also addressed via motivational feedback, relaxation prompts, and links to peer support networks.

By grounding personalization in structured, empirically informed personas, the system moves beyond generalized user models. This approach enables deeper individualization while maintaining scalability, making it particularly suitable for long-term care and chronic disease management where patient needs evolve over time.

## Questionnaire Methodology and Persona Generation

To create clinically relevant and context-aware personas, we designed a structured questionnaire aimed at capturing the multidimensional characteristics of patients living with COPD. The objective was to gather empirical data that could be mapped onto the Blueprint Personas framework and used to drive the personalization mechanisms of the intelligent agent.

The questionnaire is divided into four thematic sections: (1) demographic information, (2) lifestyle and daily habits, (3) clinical condition and use of digital tools, and (4) expectations regarding digital health support. This structure is intended to ensure the collection of both objective and subjective data essential for modeling behavior and preferences.

Demographic variables such as age, educational level, occupation, and living situation help contextualize interaction preferences and accessibility needs. Lifestyle-related questions explored patterns in diet, physical activity, social engagement, and daily routines, offering insights into potential barriers to adherence or technology adoption.

Clinical data will be collected with a focus on self-reported symptoms, disease progression, comorbidities, and previous exposure to technological tools for self-management. This information is critical for understanding the user’s level of autonomy, familiarity with digital interfaces, and openness to adopting AI-driven solutions.

The final section of the questionnaire investigates patients’ expectations regarding the digital assistant, such as desired functionalities, preferred communication style, and perceived barriers or motivators for trust.

Question types include a combination of multiple-choice, Likert-scale, and matrix formats to balance precision with user engagement. Several items were adapted from validated instruments such as PANAS (Positive and Negative Affect Schedule) [23] and SMART-Q [24], ensuring both reliability and relevance in capturing affective and behavioral dimensions.

The collected responses will be used to match each participant to the most representative persona within the Blueprint framework. This mapping process serve as the foundation for configuring the system’s personalization logic, including agent tone, frequency of prompts, and selection of intervention strategies. In future iterations, the matching process may be enhanced through rule-based decision trees or clustering techniques to support real-time persona adaptation.

By grounding personas in empirical data, this approach ensures that personalization is not only theoretically grounded but also pragmatically aligned with real-world needs and constraints. This alignment strengthens the relevance and effectiveness of the digital assistant, enabling it to function as a trusted and adaptive support tool in chronic disease management.

### How to Simulate a Mapping to Blueprint Personas

To implement the persona mapping process, we developed a structured methodology that translates questionnaire responses into ontological attributes. Each response is semantically annotated and matched to predefined variables used to instantiate the most representative Blueprint Persona. When a perfect match is not possible, the system uses a rule-based decision tree and clustering strategies to select the closest fit, while keeping track of mismatches for possible adaptation. This mapping logic is described in detail in our companion work [25], where we illustrate how individual features can be transformed into cognitive agent representations. This enables the system to flexibly update the assigned persona at runtime, mitigating the risk of user-model mismatch and improving long-term personalization.

To address the challenge of validating our mapping strategy in the absence of real-world patient data, we generated a synthetic dataset of 100 individuals using an LLM. Each simulated subject is affected by COPD, but with different severity levels or comorbidities (e.g., mild COPD, COPD with cardiovascular risk, or frequent exacerbations). This variation allows us to test personalization rules in a clinically homogeneous but demographically and behaviorally diverse population.

Each subject profile includes attributes such as age, gender identity, digital literacy, health goals, support network, trust level, and preferred robot usage. Based on this information, the system automatically assigns each profile to one of the twelve Blueprint Personas using a rule-based mapping process derived from questionnaire semantics and persona archetypes.

This mapping activates a predefined configuration of interaction tone, support priorities, and intervention strategies. For instance, a patient with COPD and frequent exacerbations, aged 86, with low digital literacy and no support network, is mapped to Maria (84), triggering high-frequency monitoring, empathetic tone, and simplified interface.

By anchoring all profiles around a single chronic disease, the simulation enables focused testing of how psychosocial and behavioral traits influence personalization in care delivery. The complete dataset, including all simulated profiles and their assigned personas, is available for inspection online.^1^

## System Architecture

**Fig. 2:**
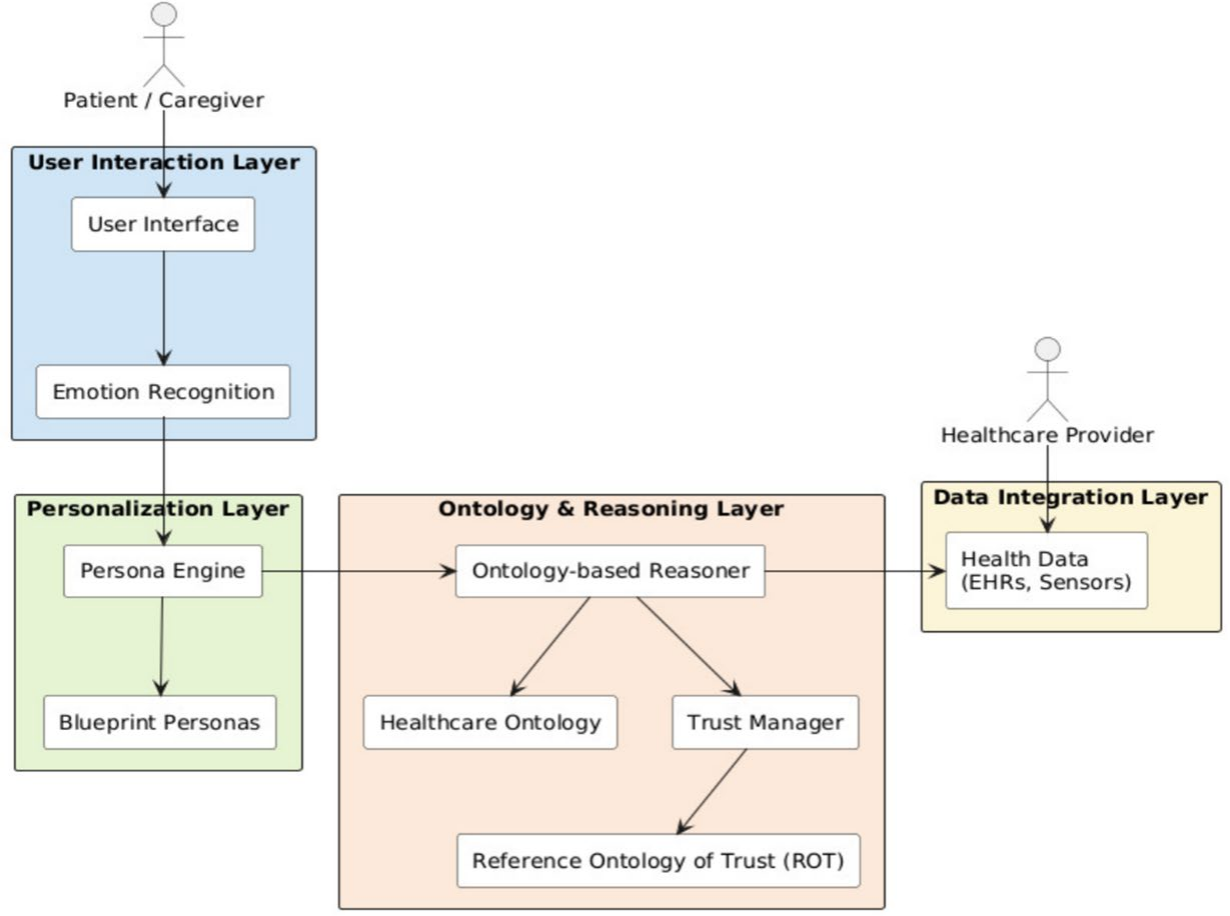
The system is structured into four main layers: the User Interaction Layer manages multi-modal communication and emotion detection; the Personalization Layer adapts content using Blueprint Personas; the Ontology and Reasoning Layer infers actions and calibrates trust using the ROT; and the Data Integration Layer enriches reasoning with live health data and clinical records

As illustrated in Fig. 2, the architecture of the proposed digital health system is structured into four functional layers that cooperate to deliver personalized, context-aware, and trustworthy support to patients and caregivers. These layers, User Interaction, Personalization, Ontology & Reasoning, and Data Integration, are designed to ensure modularity, scalability, and alignment with the principles of transparent and ethical AI in healthcare.

The **User Interaction Layer** serves as the interface between the user and the digital agent. It supports natural communication channels such as voice and touch, and includes emotion recognition mechanisms that adapt interactions based on the user’s affective state. This layer captures both explicit commands and implicit emotional cues, enabling the system to provide empathetic and responsive support. It ensures that users receive guidance in a tone, modality, and timing appropriate to their cognitive and emotional condition.

The **Personalization Layer** manages the instantiation and use of Blueprint Personas. These personas are generated from structured patient data and represent archetypes capturing clinical, behavioral, and psychosocial profiles. When a patient engages with the system, the Persona Engine identifies the most representative persona and activates corresponding personalization parameters. These parameters influence content selection, agent tone, pacing, and intervention priorities. The use of personas allows for scalable personalization while maintaining individual sensitivity to user needs.

The **Ontology & Reasoning Layer** is responsible for interpreting user needs and context through symbolic reasoning. It integrates healthcare ontologies with domain-specific rules to support explainable and flexible decision-making. At the core of this layer is an ontology-based reasoner that draws inferences based on persona attributes, current context, and system knowledge. Embedded in this layer is the Trust Manager, which uses the ROT to assess and recalibrate the system’s behavior based on evolving trust indicators. This enables the system to adjust feedback frequency, assertiveness, and recommendation strategies in a transparent and justifiable manner.

The **Data Integration Layer** connects the system with external data sources, including electronic health records (EHRs), wearable devices, and environmental sensors. It serves as a real-time monitoring backbone, ensuring that the system remains informed by the user’s current physiological state and care context. This layer also facilitates collaboration with healthcare providers, allowing clinical teams to validate and update patient information. The integration of live data ensures that recommendations remain grounded, adaptive, and clinically relevant.

Although direct access to EHRs is often limited by privacy and institutional barriers, our approach supports semantic interoperability through alignment with healthcare ontologies and standardized data exchange formats such as HL7 FHIR^2^, a widely adopted standard for structured health data exchange that facilitates integration with clinical systems and ontology-based reasoning. When EHR access is restricted, the system can rely on proxy data (e.g., questionnaires, sensor input, user feedback) to approximate personalization logic, with future integration to be validated through healthcare partnerships.

The interaction among these layers forms a coherent pipeline, in which user input is continuously processed, interpreted, and acted upon with semantic consistency and ethical transparency. The modularity of the architecture enables the integration of new ontologies, sensor data streams, and personalization templates without requiring structural redesign. Overall, this system is designed to evolve alongside user needs, technological advances, and care protocols, making it suitable for long-term deployment in dynamic healthcare environments.

**Fig. 3:**
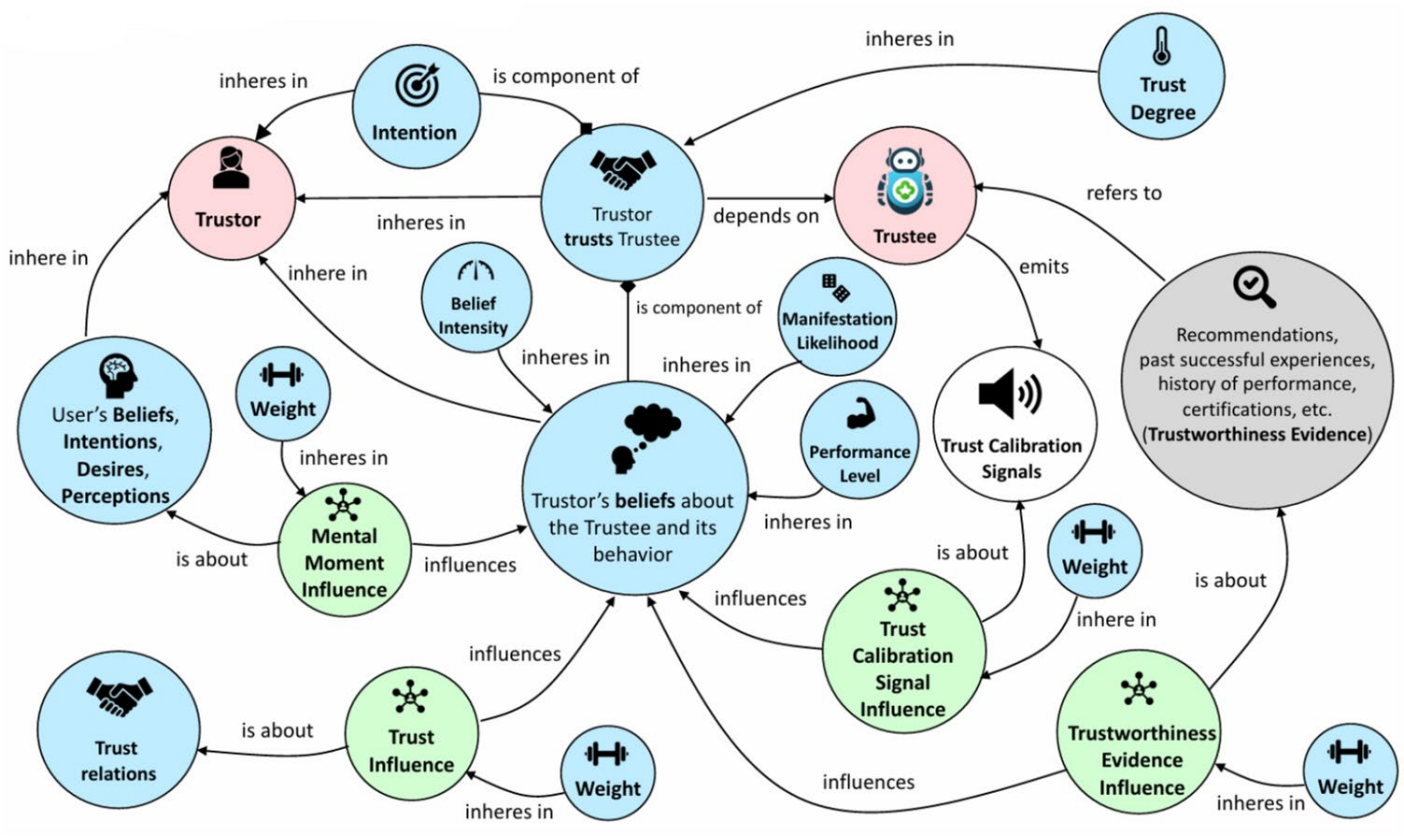
Conceptual model of ROT. The diagram illustrates the components and influences involved in the trust formation process between a Trustor and a Trustee, including beliefs, intentions, trust calibration signals, and evidential factors. The model supports dynamic trust reasoning by representing both cognitive and contextual dimensions, enabling adaptive system behavior in AI-driven healthcare scenarios

## Ontologies and Trust Reasoning

Ontologies play a central role in enabling semantic interoperability, transparent reasoning, and structured knowledge representation in healthcare AI systems. In the proposed architecture, ontologies are used to encode patient knowledge, system rules, and trust dynamics, forming the backbone of context-aware and explainable personalization. This section outlines how ontologies drive reasoning in the system and describes the integration of a formal trust model through ROT.

### Ontology-driven Personalization

Ontologies, originally a philosophical construct, have become fundamental in computer science for modeling structured knowledge domains [26]. In artificial intelligence, they offer a shared vocabulary to describe entities, attributes, and relationships, enabling systems to reason, infer, and communicate with both humans and machines [27].

Within this work, ontologies are employed to formalize the knowledge embedded in Blueprint Personas and to operationalize personalization. Each persona is modeled as a structured profile comprising health conditions, behavioral traits, needs, and contextual factors. The ontology encodes this information alongside caregiver tasks, clinical pathways, and assistive interventions, creating a unified framework for reasoning. This formalization allows the system to dynamically adapt to user needs, prioritize care actions, and suggest recommendations based on real-time data or historical patterns.

The ontology also acts as a bridge between heterogeneous stakeholders, patients, caregivers, and healthcare providers, by fostering semantic interoperability. Integration with external systems, such as EHRs, is streamlined through standardized concepts, ensuring that user models remain coherent and up-to-date. Furthermore, the reasoning capabilities enable the system to manage incomplete information by drawing logical conclusions from available evidence, improving both robustness and responsiveness [28].

By grounding personalization in ontology-based modeling, the system transcends static rule sets and embraces adaptive behavior that reflects real-world complexity and user diversity [29].

A key benefit of this ontology-driven approach is the AI system’s ability to reason and make inferences. For example, if a caregiver is unavailable, the system could infer which other caregivers are qualified to step in based on their roles and skill sets, automatically adjusting care plans accordingly. The efficient reasoning element and transparency of a system based on symbology also offer the possibility to modify in a targeted way any system elements, offering the patient an even more personalized experience [30]. Additionally, the system can manage incomplete information by drawing logical conclusions from known facts, ensuring that it can still provide useful advice or actions even when certain data points are missing.

### The Reference Ontology of Trust (ROT)

To ensure ethical alignment and sustained engagement, the system incorporates a formal trust model through the ROT [7]. ROT conceptualizes trust as a dynamic mental construct involving beliefs, expectations, past interactions, and contextual factors.

Within this model, the patient (Trustor) forms judgments about the system (Trustee) based on perceptions of capability, vulnerability, and intention. These judgments are shaped by both prior experience and real-time cues, termed Trust Calibration Signals, including message tone, system consistency, transparency, and responsiveness.

Key elements of the trust model include:

- **Trustworthiness Evidence**: Historical behavior, endorsements, and system reliability.
- **Trust Calibration Signals**: Real-time indicators such as tone of communication, delay in responses, or message clarity.
- **Mental Moment Influences**: Contextual and emotional states that alter trust sensitivity.
- **Manifestation Likelihood**: The perceived probability that the system will behave as expected.
- **Trust Degree**: A dynamic score representing current trust level, influencing system behavior.

These components are encoded in the Trust Manager, a module within the Reasoning Layer that modulates feedback strategies based on the evolving trust profile of each user. For instance, if the system detects reduced adherence or negative emotional responses, it can shift from assertive reminders to more supportive language and offer explanatory prompts.

By integrating ROT, the system does not assume static user trust but actively calibrates it over time. This aligns agent behavior with user expectations, preserves autonomy, and improves transparency, which is particularly critical in sensitive healthcare scenarios.

ROT provides a robust foundation for ethical and adaptive interaction by making trust both interpretable and computationally actionable. It supports risk-aware reasoning and explainability, thereby strengthening the agent’s role as a reliable and empathetic assistant, as shown in Fig. 3.

## Application Scenario: COPD Patient Support

To illustrate the functionality and applicability of the proposed architecture, we present a simulated use case involving a fictional patient, Maria, a 72-year-old woman diagnosed with COPD. Maria has limited digital literacy, lives alone, and occasionally receives visits from a nurse. She struggles with respiratory symptoms, fatigue, and social isolation.

Upon initial onboarding, Maria completes a structured questionnaire. Based on her responses, the system assigns her a Blueprint Persona characterized by physical frailty, low technology affinity, and emotional vulnerability. This persona configures the system’s personalization parameters, which include interaction pacing, communication style, trust sensitivity, and engagement strategy.

Maria interacts with the Personal Assistant Agent (PAA) via a tablet equipped with a voice-based interface. The *User Interaction Layer* enables natural, low-effort communication. Based on emotion recognition and interaction history, the agent adapts tone and complexity, slowing speech, simplifying instructions, or offering encouragement.

The *Personalization Layer* uses the assigned persona to determine how the agent should prioritize content and interventions. In Maria’s case, reminders are gentle, time-based, and infrequent to reduce cognitive load. The agent also avoids push notifications during stress-detected moments and incorporates empathy-driven phrases in the dialogue.

The *Ontology and Reasoning Layer* continuously interprets contextual data. When air quality deteriorates, the agent cross-references Maria’s respiratory sensitivity and emits a proactive alert: *“The air quality today is poor, please consider staying indoors and using your air purifier.”* This reasoning is based on semantic inference from environmental data and medical thresholds defined in the ontology.

The *Data Integration Layer* supplies the system with real-time updates from wearable devices (e.g., pulse oximeter) and electronic health records. This information is used by the Reasoner to detect patterns such as worsening symptoms or missed medication, prompting the agent to intervene or escalate alerts to the healthcare provider.

Trust calibration is dynamically managed through the *Reference Ontology of Trust*. For instance, if Maria ignores several recommendations, the system interprets this as potential trust erosion and adjusts accordingly, offering explanations, reducing directive language, and reinforcing positive interactions. In contrast,, high compliance reinforces a consistent and confident communication style.

The scenario demonstrates how the system integrates user profiling, semantic reasoning, and adaptive trust mechanisms to provide contextualized, personalized, and ethically grounded support for chronic disease self-management. It highlights the system’s capacity to evolve with the user, maintain engagement, and deliver meaningful assistance in daily care.

### Symbolic Implementation of Trust Reasoning

To operationalize the scenario described above, we implemented a symbolic reasoning component using Answer Set Programming (ASP). The agent observes the user’s behavior and uses ASP rules to infer their trust level. Based on this inference, it dynamically adjusts the tone of communication and delivers context-sensitive recommendations.

Listing 1 presents the ASP rules that define this reasoning process. The user behavior is interpreted (e.g., ignoring or following a recommendation), and a corresponding trust level is inferred. Based on this, the system selects a personalized tone and generates a relevant health recommendation. Logical constraints ensure consistency between different trust states.
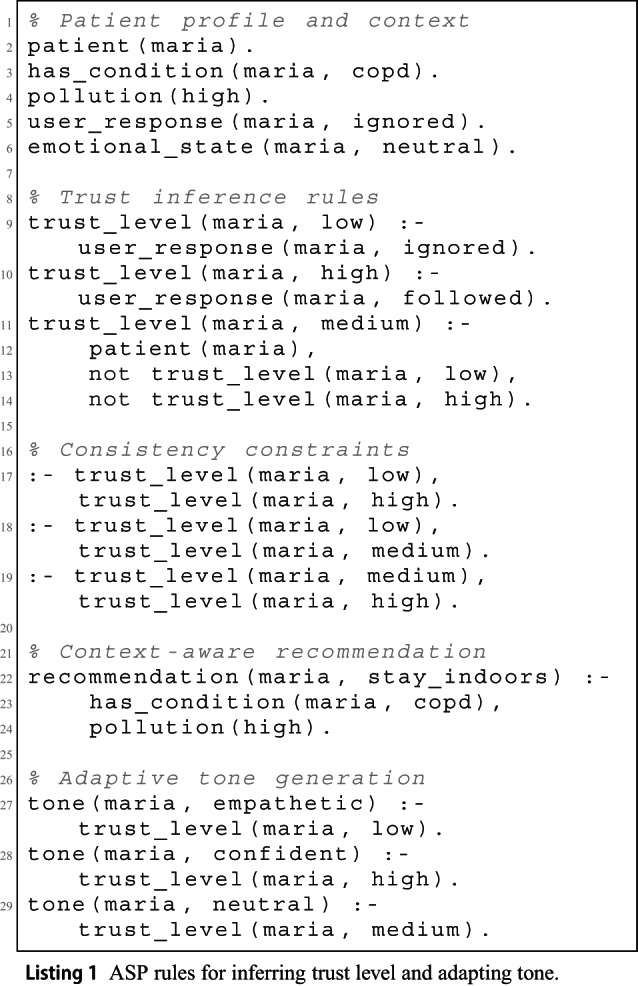


To support semantic reasoning, the system integrates a lightweight ontology that encodes patient profiles, medical conditions, and trust-based interaction patterns. Listing 2 shows a fragment in Turtle format, where the patient :Maria is linked to the class :COPD and associated with a trust level that affects tone and alert style.
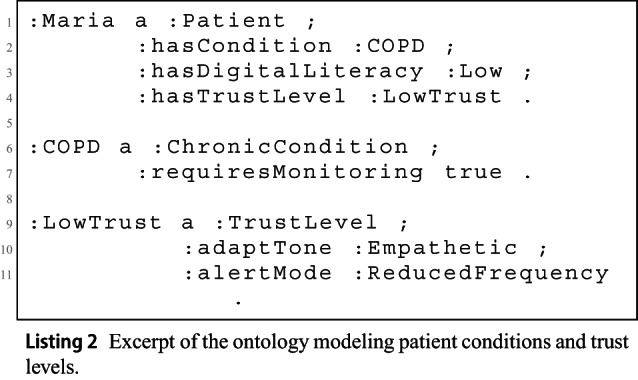


While the symbolic rules define static mappings, the system also includes a dynamic trust calibration mechanism, used in runtime deployments. Listing 3 provides a pseudocode sketch of the trust update function, which adjusts the trust score based on user feedback. The tone is then selected according to the current trust range.
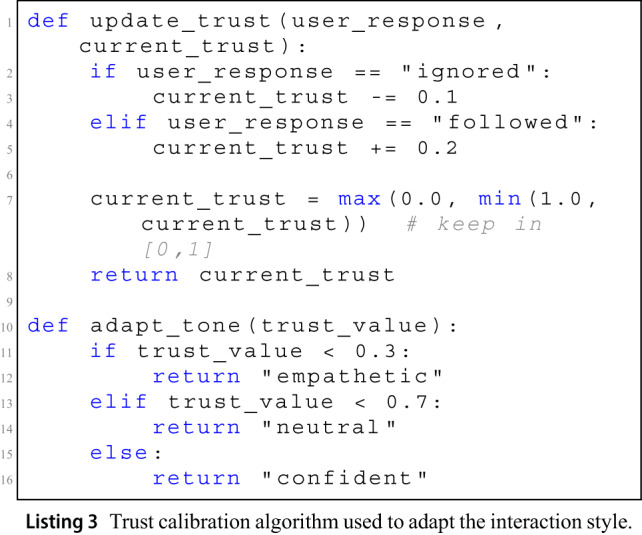


This modular implementation enables explainable and adaptable agent behavior, grounded in both symbolic rules and empirical user feedback.

While emotion recognition from speech remains a nontrivial challenge, especially in spontaneous conversation and across linguistic or age-specific variations, recent experimental work conducted within our team confirms the feasibility of lightweight and interpretable affective models. In particular, Santomaggio [31] implemented and validated a supervised pipeline using openSMILE and GeMAPS descriptors on Italian and English speech datasets (EMOVO and RAVDESS), training SVM classifiers to identify emotional states with promising results. Techniques such as noise injection and augmentation were applied to address data sparsity and improve robustness, offering a pragmatic path toward emotion-aware digital assistants also suited to older users.

## Expected Evaluation and Impact

To support future validation, we identify a set of evaluation criteria and expected outcomes grounded in prior literature on digital health, trust modeling, and user-centered AI systems.

User trust will be assessed through semantic indicators derived from the ROT and a 5-point Likert questionnaire. Usability will be measured using the System Usability Scale (SUS), a well-established tool for assessing perceived ease of use. Therapeutic adherence will be monitored as the percentage of interventions successfully followed by the user after system prompts.

In addition, key performance indicators (KPIs) include:

- **User Trust Level:** inferred semantically and complemented by user feedback.
- **Perceived Usability:** collected via SUS scores after interaction cycles.
- **User Satisfaction:** evaluated through post-interaction Likert questionnaires.
- **Therapeutic Adherence:** computed as the ratio of accepted vs. ignored system prompts.
- **Interaction Appropriateness:** measured as the alignment between trust level and agent tone.

The agent’s symbolic reasoning component supports explainable tracking of these dimensions, particularly trust, by linking user behavior to semantic constructs such as calibration signals and inferred mental states.

Based on related studies in socially assistive technology and adaptive eHealth agents, we expect the integration of dynamic trust calibration and personalized reasoning to enhance long-term engagement, increase perceived empathy, and improve user-system alignment. Moreover, the use of Blueprint Personas is expected to support inclusive, context-aware design and positively impact usability and adherence across diverse user profiles.

**Table 1:** Evaluation Plan and Expected Outcomes

Metric	Measurement Method	Expected Outcome
User Trust Level	Semantic indicators (ROT) + 5-point Likert scale	70% report medium-to-high trust after 2 weeks
Perceived Usability	System Usability Scale (SUS)	SUS score $\ge 75$ (above-average usability)
User Satisfaction	Post-interaction Likert questionnaires	80% positive feedback on tone and clarity
Therapeutic Adherence	Ratio of accepted vs. ignored system prompts	$\ge 65\%$ adherence rate after initial onboarding
Interaction Appropriateness	Match between inferred trust and system tone	$\ge 85\%$ alignment between tone and trust level

While empirical validation is planned as part of future work, we outline a hypothetical evaluation plan to demonstrate how the effectiveness of the system could be measured. Table 1 summarizes the key metrics, proposed measurement methods, and expected outcomes based on prior literature and anticipated system behavior. These values reflect targets for upcoming pilot studies and simulations involving user interaction with the intelligent agent over short-term evaluation cycles (e.g., two weeks of guided usage).

## Ethical Considerations

The design of AI-driven health support systems raises critical ethical challenges, particularly in contexts involving chronic disease management and vulnerable populations such as elderly patients [32]. The proposed system addresses these concerns through a human-centered and transparent approach, embedding ethical principles throughout the architecture.

**Privacy and Data Protection**: The system is developed with a privacy-by-design approach. All personal data, including health status, behavioral patterns, and emotional cues, are processed securely and stored in compliance with applicable data protection regulations. Users have control over their data sharing preferences, and consent is explicitly obtained for data integration with external systems (e.g., EHRs).

**Transparency and Explainability**: To foster user trust, the system provides interpretable feedback and justifications for its actions, especially when recommending behavioral changes or alerting to potential risks. Ontology-based reasoning enables the system to generate traceable logic paths that can be reviewed by healthcare professionals or the user.

**Equity and Accessibility**: By leveraging Blueprint Personas, the system promotes inclusive design that accounts for diverse cognitive, social, and cultural profiles. This ensures that the agent does not reinforce biases or exclude underrepresented populations in digital health.

**Trust and Autonomy**: The integration of ROT ensures that trust is calibrated over time and based on user interaction, rather than assumed. The system never overrides user autonomy and is designed to assist, not replace, human decision-making.

In summary, ethical concerns are addressed not as external constraints but as design criteria integral to the system’s logic, structure, and interaction model.

## Conclusion

This work presents a modular architecture for trust-aware digital health systems that integrates Blueprint Personas, ontology-based reasoning, and dynamic trust modeling. The proposed system aims to provide explainable, ethically aligned, and personalized support for chronic disease management, especially in the context of COPD. At its core, the architecture leverages semantic layers to align user modeling, reasoning, and adaptive interaction strategies.

Ontologies play a central role in structuring and interpreting the relationship between patients, caregivers, and digital agents. Their human- and machine-readable nature supports transparency and interoperability, enabling meaningful knowledge exchange. The incorporation of the Reference Ontology of Trust (ROT) introduces a principled approach to modeling and calibrating trust within human-agent interactions.

To demonstrate the system’s potential, we simulated a scenario involving a fictional COPD patient. This case study illustrated how the system adapts interaction tone and support strategies based on real-time contextual triggers, user profiles, and ethical principles such as autonomy, transparency, and privacy.

### Limitations

Despite its promising structure, the proposed system is still under development and subject to several limitations. First, the reasoning and personalization mechanisms have not yet been validated through clinical deployment or longitudinal user studies. The current validation is based on simulated personas generated via large language models, which, while structurally diverse, do not reflect the full behavioral and emotional complexity of real patients.

Second, while emotion recognition and trust modeling components are in place, they rely on offline pipelines and simplified interaction contexts. Their performance in spontaneous, multi-turn dialogues, particularly with older or vulnerable populations, remains untested.

Third, integration with EHRs is currently theoretical due to access constraints, and real-time data flows from wearables and clinical systems are yet to be implemented.

### Future Work

Future developments will address these limitations through four main directions. First, we plan to conduct empirical validation through user studies involving both clinicians and older adults to assess usability, engagement, and emotional alignment. Second, we will expand the ROT framework with more granular trust dimensions, including adaptability to cultural and generational variations.

Third, we aim to operationalize interoperability with wearable devices and explore privacy-preserving strategies for integrating with clinical platforms under ethical supervision. Finally, the persona assignment pipeline will be refined by incorporating feedback-driven adaptations and hybrid symbolic-ML methods.

Ultimately, this research contributes a conceptual and technical foundation for AI-driven digital health agents that promote trust, personalization, and transparency in chronic care support.

## Data Availability

No datasets were generated or analysed during the current study.

## Footnotes

1 https://github.com/AlinaVozna/blueprint-personas-copd.git

2 https://www.hl7.org/fhir/
